# Multidisciplinary consensus-based statement on the current role of middle meningeal artery embolization (MMAE) in chronic SubDural hematoma (cSDH)

**DOI:** 10.1016/j.bas.2024.104143

**Published:** 2024-11-19

**Authors:** J. Bartek, A. Biondi, V. Bonhomme, L. Castellan, G. Catapano, M. Cenzato, G. Di Nuzzo, E. De Robertis, F. Giordano, C. Iaccarino, Z. Kulcsar, M.A. Möhlenbruch, A. Raabe, F. Rickard, C.S. Romero, T. Schubert, Shipway D, C. Sicignano, M. Muto

**Affiliations:** EANS Delegate, Dep. of Neurosurgery, Karolinska University Hospital, Stockholm, Sweden; WFITN Delegate, Department of Interventional Neuroradiology, Besançon University Hospital, F-25000, Besançon, France; Laboratoire de Recherches Intégratives en Neurosciences et Psychologie Cognitive, Université Bourgogne Franche-Comté, F-25000, Besançon, France; ESAIC Delegate, Department of Anesthesia and Intensive Care Medicine, Liege University Hospital, Liege, Belgium; Anesthesia and Perioperative Neuroscience Laboratory, GIGA-Consciousness, GIGA-Research, Liege University, Liege, Belgium; AINR Delegate, Neuroradiology Department, San Martino Hospital, Genoa, Italy; SINCh Delegate, Neurosurgery Unit, Ospedale Del Mare, Naples, Italy; EANS Delegate, Dept. of Neurosurgery, Niguarda Hospital, Milan, Italy; SINCh Delegate, Neurosurgery Unit, Ospedale Del Mare, Naples, Italy; ESAIC Delegate, Section of Anaesthesia, Analgesia and Intensive Care, Department of Medicine and Surgery, University of Perugia, Italy; ESNR Delegate, Diagnostic and Interventional Neuroradiology, Cardarelli Hospital, Naples, Italy; NTC-WFNS Delegate, Advisory Group of the Neurotraumatology Committee of the WFNS, Neurosurgery, School of Neurosurgery, University of Modena and Reggio Emilia, Italy; Neurosurgery Division, University Hospital of Modena, Modena, Italy; Neurosurgery Unit, AUSL RE IRCCS, Reggio Emilia, Italy; ESMINT Delegate, Department of Neuroradiology, Clinical Neuroscience Center, University Hospital of Zurich, Zurich, Switzerland; ESNR Delegate, Neuroradiology, Heidelberg University Hospital, Heidelberg Germany; EANS Delegate, Department of Neurosurgery, Inselspital Bern, Bern, Switzerland; EuGMS Delegate, Consultant Geriatrician and Perioperative Physician, North Bristol NHS Trust, Bristol, UK; ESAIC Delegate, Department of Anaesthesia and Intensive Care Medicine, University General Hospital, European University of Valencia, Spain; ESMINT Delegate, Department of Neuroradiology, Clinical Neuroscience Center, University Hospital of Zurich, Zurich, Switzerland; EuGMS Delegate, Consultant Physician & Perioperative Geriatrician, North Bristol NHS Trust, Bristol, UK; Chair, British Geriatric Society Perioperative Medicine Group, UK; Honorary Senior Clinical Lecturer, University of Bristol, UK; AINR Delegate, Neuroradiology, Ospedale Del Mare, Naples, Italy; ESNR Delegate, Diagnostic and Interventional Neuroradiology, Cardarelli Hospital, Naples, Italy

**Keywords:** Chronic subdural hematoma, Endovascular, Middle meningeal artery embolization, Neurosurgery, Anaesthesia, Geriatrics

## Abstract

**Introduction:**

Middle Meningeal Artery Embolization (MMAE) in patients with chronic SubDural Hematoma (cSDH) is a novel treatment approach, albeit the specific role of MMAE in the treatment of cSDH is not yet defined.

**Research question:**

The aim of this work is to provide a consensus-based statement from a multidisciplinary panel on the current role of MMAE in patients with cSDH.

**Materials and methods:**

A literature search was performed using the keywords MMAE and cSDH. Based on the available published data, the panel was asked if a consensus could be reached on the role of MMAE in both de novo as well as in recurrent cSDH.

**Results:**

The panel reached a consensus on the current role of MMAE in both de novo- and recurrent cSDH, as well as in patients on antithrombotics and those with coagulopathy. MMAE should be *considered* in the following scenarios:

1) As “stand-alone” treatment in de novo cSDH requiring intervention but where surgery is prevented due to either coagulopathy or in patients on antithrombotics in whom the risk of suspension is considered unacceptably high,
2) as “stand-alone” treatment in recurrent cSDH requiring intervention but where surgery is prevented due to either coagulopathy or in those on antithrombotics in whom the risk of suspension is considered unacceptably high and
3) as “adjunct to surgery” in all recurrent cSDH.

**Discussion and conclusion:**

This statement is to be considered an expert consensus opinion of delegates representing key international medical societies of specialists involved in the care of cSDH patients.

## 1 Introduction

The incidence of patients with chronic SubDural Hematoma (cSDH) is increasing as the population of Europe ages. cSDH has become the most frequent neurosurgical diagnosis in developed countries but is also of increasing global health- and socio-economic importance as populations age globally. The growing interest in cSDH is also reflected in the increased activity in cSDH research, with emphasis on improving current surgical treatment as well as introducing new treatment modalities (Edlmann et al., 2020). While the new pharmacological interventions so far have shown mixed results, the improvements of surgical technique have reduced recurrence rates, complication frequency, as well as length of inpatient hospital stay. This not only improves patient outcomes but also reduces the health economic burden of cSDH (Bartley et al., 2023; Raj et al., 2024; Sjavik et al., 2017; Zolfaghari et al., 2021; Hjortdal et al., 2023, 2024). Nevertheless, at the same time, older patients with cSDH are frequently co-medicated with antiplatelets or anticoagulants (here forth antithrombotic) therapy, making them particularly at risk of adverse events following surgery and reflecting the need for novel treatment options.

**Fig. 1:**
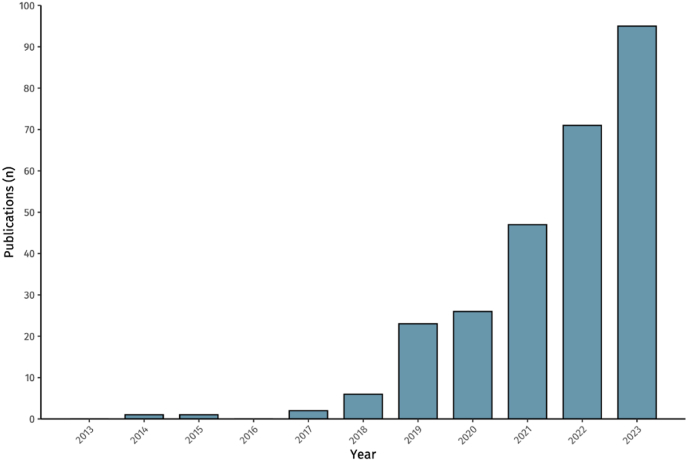
Number of publications on MMAE over the past years.

Recently, the novel endovascular treatment option of Middle Meningeal Artery Embolization (MMAE) has emerged. While this treatment has shown promising results in observational datasets, data from randomised clinical trials is so far lacking, although numerous randomised trials are underway and some have already presented their preliminary findings (EMBOLISE abstract; MAGIC MT abstract https; STEM abstract https). As such, the research activity in this field is growing, as reflected by the increasing number of publications (Fig. 1).

The abrupt expansion in published data has resulted in some centres adopting MMAE as adjunct- or stand-alone standard treatment in *all* cases of cSDH (Rudy et al., 2023); while other centres have not yet adopted MMAE for a variety of reasons including, insufficient experience and/or resources, and/or financial approval to utilise this technique at the present time.

Moreover, there remains no agreed consensus on the indications for MMAE. Therefore, clinical practice remains inconsistent and heterogeneous between, and even within individual regions, as highlighted by recent discussions within the European Association of Neurosurgical Societies (EANS), European Society for Minimally Invasive Neurological Therapy (ESMINT) and European Society of Neuroradiology (ESNR).

Therefore, the key European and other international societies representing clinicians who regularly participate in the treatment of patients with cSDH have formed a consensus working group, with the aim of providing the first multidisciplinary consensus-based statement on the use of MMAE in cSDH based on the current available literature.

## 2 Methods

### 2.1 Aim

To provide a consensus-based statement from a multidisciplinary panel on the current role of MMAE in cSDH.

### 2.2 Panel composition

To assure a multi-disciplinary approach and round table discussion, delegates from key societies of specialists involved in the care of cSDH patients were selected by their respective societies, based on their expertise within the field of cSDH management. The societies represented were.

1. European Association of Neurosurgical Societies (EANS)
2. European Society for Minimally Invasive Neurological Therapy (ESMINT)
3. European Society of Neuroradiology (ESNR)
4. European Geriatric Medicine Society (EuGMS)
5. European Society of Anaesthesiology and Intensive Care (ESAIC)
6. Italian Society of Neurosurgery (SINCh)
7. Italian Association of Diagnostic and Interventional Neuroradiology (AINR)
8. World Federation of Interventional and Therapeutic Neuroradiology (WFITN)
9. Neurotraumatology Committee (NTC) of the World Federation of Neurosurgical Societies (WFNS)

### 2.3 Literature search

An English language literature search was performed using the PubMed, Embase, Scopus, and Web of Science databases to identify articles published until March 30th, 2024. The study search was restricted to data obtained in humans and conducted using the following key words with their variations: “Middle meningeal artery embolization”, “chronic subdural hematoma”, “chronic subdural hematoma treatment”, “chronic subdural hematoma management”, “chronic subdural hematoma embolization”. The search resulted in 451 articles. The title and abstract of potentially relevant studies were screened for appropriateness before retrieval of the full article by two reviewers (C.S. and F.G.) and disagreements were discussed with a third reviewer (M.M.) to reach a consensus. The full-published reports of the abstracts selected by the reviewers were retrieved and the same reviewers independently performed a second-step selection based on the inclusion criteria; disagreements were resolved by consensus. Furthermore, in accordance with PRISMA guidelines, the bibliographies of retrieved articles were manually reviewed to identify additional items meeting inclusion criteria.

### 2.4 Consensus work-flow

The round-table meeting took place on the April 16, 2024. Prior to the meeting, all delegates were provided with the results of the literature search and asked to critically assess the listed publications and an online meeting was held to agree on the consensus work-flow. Further, delegates of the respective societies were asked to prepare a short statement/presentation on their view of the use of MMAE in cSDH. After the presentations, discussion on the role of MMAE in de novo- and recurrent cSDH took place.

Initially, the panel decided to define certain terms to be able to go forward with a joint statement. The terms needing defining were “cSDH needing intervention” and “recurrent cSDH”. "cSDH needing intervention" = cSDH that requires active management with either surgery and/or MMAE based on the patients’ symptoms and/or radiological features. "Recurrent cSDH" = a cSDH needing renewed intervention. The consensus process was documented by the independent Editorial Assistant and Facilitator of the group (A.C.). Following the round-table meeting, an additional 5 online meetings were organised, with the whole process taking 3 months.

## 3 Results

The panel reached a consensus on the current role of MMAE in both de novo cSDH and recurrent cSDH, as well as in patents on antithrombotics and those with coagulopathy. MMAE should be considered in following 3 scenarios.

1) As *“stand-alone”* treatment in de novo cSDH requiring intervention but where surgery is prevented due to either coagulopathy or in those on antithrombotics in whom the risk of suspension is considered unacceptably high,
2) As *“stand-alone”* treatment in recurrent cSDH requiring intervention but where surgery is prevented due to either coagulopathy or in those on antithrombotics in whom the risk of suspension is considered unacceptably high
3) As *“adjunct to surgery”* in recurrent cSDH.

Further, it is important to state that in patients with substantial mass effect/neurological deficits, surgical intervention may be indicated even though these are on antithrombotics in whom the risk of suspension is very high. In these patients, MMAE as adjunct to surgery should be considered in order to allow for early resumption of antithrombotics.

**Fig. 2:**
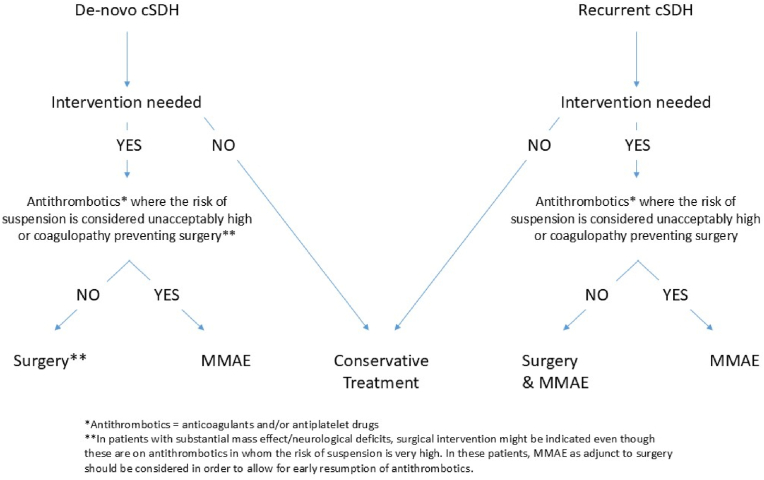
Consensus flow-chart of MMAE use in de novo- and recurrent cSDH.

Please see Fig. 2 for the flow chart of the above statement.

## 4 Discussion

While surgery is the mainstay of cSDH treatment, variations in treatment practice and outcome reporting have resulted in heterogenous data being reported, with seemingly high recurrence frequencies as well as non-negligible rates of postoperative complications and mortality.

Nevertheless, modern-era data using state-of-the-art surgical treatment protocols report a recurrence rate of approximately 10–20%, few serious adverse events, and a negligible mortality rate; higher mortality rates are most often associated with age and multimorbidity, as opposed to the surgery itself (Bartley et al., 2023; Raj et al., 2024; Bartek et al., 2017a, 2017b; Lodewijkx et al., 2023). Although these results are encouraging, there is still room for further improvement in the treatment and associated outcomes of these patients. Furthermore, special attention needs to be devoted to the increasing number of patients medicated with antithrombotics, who have an augmented risk of cSDH recurrence, while withdrawal of antithrombotics for a prolonged time increases their thromboembolic risk (Zanaty et al., 2019; Murthy et al., 2020; Fornebo et al., 2017).

Recently, it has been recognised that inflammation plays a key role in the pathogenesis of cSDH (Edlmann et al., 2017). As such, an important number of trials attempting to use different pharmacotherapies (mainly anti-inflammatory) as stand-alone or adjunctive treatments of cSDH has occurred, albeit these have so far ended up with mixed results, with some demonstrating efficacy while having an unfavourable complication profile – thus so far without any of the proposed therapies becoming standard of care (Yu et al., 2022; Hutchinson et al., 2020; Wang et al., 2021; Miah et al., 2023). Similarly, cSDH is now recognised as an inflammatory cerebrovascular disease, involving neovascularisation by pathological "leaky" blood vessels, for which MMAE has emerged as a novel treatment option. The rationale behind the treatment efficacy is the targeting and obliteration of the neovasculature, from which the neomembrane has been shown to derive its blood supply via the middle meningeal artery (MMA). By targeting the MMA, the goal is to disrupt the pathogenesis by reducing the blood supply to the neovasculature (Fiorella and Arthur, 2019). MMAE has gained popularity due to the promising reports of its efficacy in retrospective datasets (Haldrup et al., 2020; Omura and Ishiguro, 2023; Desir et al., 2023; Rojas-Villabona et al., 2023; Dian et al., 2021; Chen et al., 2023, 2024; Ironside et al., 2021). Some centres with easy access to neurointerventional procedures and adequate insurance coverage for patients have adopted it as a standard of care (Rudy et al., 2023), while other centres have not yet adopted MMAE, due to the lack of financial approval, consensus regarding its indications and the current absence of prospective randomised data. For instance, in the UK, the National Institute for Health and Care Excellence (NICE) have advised that MMAE for chronic subdural haematomas should be used only in a research context (https://www.nice.org.uk/guidance/ipg779.). Due to this wide variation in practice, the five key European and four other societies of specialists involved in the management of cSDH patients have decided to propose this consensus statement to guide practice whilst we await the results from the multiple ongoing randomised controlled trials - please see Table 1 for an overview of the current randomised ongoing trials.

**Table 1:** Current randomised trials on eMMA in cSDH.

Title	Primary Location	Status	Registration
Dartmouth Middle Meningeal Embolization Trial (DaMMET)	Lebanon, United States	Completed	NCT04270955
Middle Meningeal Artery Embolization for Treatment of Chronic Subdural Hematoma	New York, United States	Completed	NCT03307395
The SQUID Trial for the Embolization of the Middle Meningeal Artery for Treatment of Chronic Subdural Hematoma (STEM)	Phoenix, United States	Completed	NCT04410146
Middle Meningeal Artery Embolization for the Treatment of Subdural Hematomas With TRUFILL® n-BCA (MEMBRANE)	New York, United States	Active, not recruiting	NCT04816591
Managing Non-acute Subdural Hematoma Using Liquid Materials: a Chinese Randomized Trial of MMA Treatment (MAGIC-MT)	Huanshan, China	Active, not recruiting	NCT04700345
Embolization of the Middle Meningeal Artery With ONYX™ Liquid Embolic System for Subacute and Chronic Subdural Hematoma (EMBOLISE)	Birmingham, United States	Recruiting	NCT04402632
Embolization of Middle Meningeal Artery for Subdural Hematoma in Canada (EMMA Can)	Mannitoba, Canada	Recruiting	NCT04923984
Management of CSDH With or Without EMMA- a Randomized Control Trial (EMMA-Can)	Mannitoba, Canada	Recruiting	NCT04750200
Embolization of Middle Meningeal Artery in Chronic Subdural Hematoma (ELIMINATE)	Amsterdam, Holland	Recruiting	NCT04511572
The Onyx™ Trial For The Embolization Of The Middle Meningeal Artery For Chronic Subdural Hematoma (OTEMACS)	Montpellier, France	Recruiting	NCT04742920
Swedish Trial on Embolization of Middle Meningeal Artery Versus Surgical Evacuation in Chronic Subdural Hematoma (SWEMMA)	Lund, Sweden	Recruiting	NCT05267184
Preventing Recurrences of Chronic Subdural Hematoma in Adult Patients by Middle Meningeal Artery Embolization (MEMBRANE)	Berlin, Germany	Recruiting	NCT05327933
Efficacy of a Minimally Invasive Therapy Adjuvant to the Standards of Care by Cyanoacrylate Embolization (LEADH)	Brest, France	Recruiting	NCT05374681
Endovascular Embolization for Chronic Subdural Hematomas Following Surgical Evacuation (endovascular)	Augusta, United States	Recruiting	NCT04272996
Middle Meningeal Artery Embolization for Chronic Subdural Hematoma	Missouri, United States	Recruiting	NCT04065113
Middle Meningeal Artery Embolization With Liquid Embolic Agent for Treatment of Chronic Subdural Hematoma	Mashhad, Iran	Recruiting	NCT04574843
Middle Meningeal Artery Embolization for Chronic Subdural Hematomas (STORMM)	Geneva, Switzerland	Not Yet Recruiting	NCT06163547
Middle Meningeal Artery Embolization for the Treatment of Subdural Hematomas With TRUFILL® n-BCA (MEMBRANE)	New York, United States	Active, not recruiting	NCT04816591
Puerto Rico Embolization of the Middle Meningeal Artery for the Treatment of Chronic Subdural Hematoma Trial (PREMMA)	San Juan, Puerto Rico	Not Yet Recruiting	NCT06466733
Endovascular vs Conservative Treatment in Patients With Chronic Subdural Hematomas and Mild Symptoms	Genova, Italy	Not Yet Recruiting	NCT06274580
Chronic Subdural Hematoma Treatment With Embolization Versus Surgery Study (CHESS)	Galveston, United States	Not Yet recruiting	NCT06347796
Endovascular Embolization of Chronic Subdural Hematomas After Surgery (ENCLOSURE)	Barcelona, Spain	Unknown Status	NCT05220826
Embolization of the Middle Meningeal Artery for the Prevention of Chronic Subdural Hematoma Recurrence in High Risk Patients (EMPROTECT)	Paris, France	Unknown Status	NCT04372147
Middle Meningeal Artery (MMA) Embolization Compared to Traditional Surgical Strategies to Treat Chronic Subdural Hematomas (cSDH)	Summit, United States	Unknown Status	NCT04095819

On review of the current evidence, it is apparent that MMAE is being utilised in a heterogenous range of patients with a variety of symptoms and radiological findings, as well as patients with both de novo and recurrent cSDH. The observational data can be summarized as reporting a high efficacy of MMAE in terms of limiting cSDH progression when used as “stand alone” treatment as well as reducing recurrence rates when used as an “adjunct” to surgery (Haldrup et al., 2020; Omura and Ishiguro, 2023; Desir et al., 2023; Rojas-Villabona et al., 2023; Dian et al., 2021; Chen et al., 2023, 2024; Ironside et al., 2021). The observational data from the largest systematic review including 9 studies with 1523 patients can be summarized as reporting a treatment failure in 5.6% in the MMAE group and 22.2% in the conventional management group with lower risk of surgical rescue being necessary in the MMAE group vs. the conventional management group (4.1% vs 16.1%). The complication frequency, functional outcome (mRS) and mortality did not differ between the MMAE group vs. the conventional management group – although complications such as cerebral infarctions, visual loss, facial palsy, MMA arteriovenous fistula, cerebral haemorrhage, MMA rupture and ICA dissection were observed. While the authors concluded that MMAE decreases treatment failure and the need for surgical rescue without furthering the risk of morbidity and mortality, none of the available observational datasets have so far been reported in a standardized fashion, with a risk of subjective assessment by the treatment physicians themselves, as well as a risk of selection bias when MMAE candidates were chosen (Sattari et al., 2023).

### 4.1 Use of MMAE in de novo cSDH

De novo cSDH patients can be subdivided into those needing some sort of intervention, defined as a cSDH that requires active management such as surgery and/or MMAE based on the patients’ symptoms, antithrombotic/coagulopathy status and/or radiological features versus those not needing any intervention.

With regards to the so far unpublished randomised controlled trial datasets of de novo cSDH patients treated with MMAE, both the EMBOLISE, MAGIC-MT and the STEM investigators preliminary report a superior efficacy in terms of recurrence in both the MMAE stand-alone and surgical adjunct groups of patients. Further, they all met the safety endpoints of their studies without any deaths attributed to the MMAE technique (EMBOLISE abstract https; MAGIC MT abstract https; STEM abstract https). As such, the preliminary data are indeed promising, albeit the specific subgroup analysis of these cohorts in terms of symptoms, radiological features and comorbidities are pending. We need to await these, as well as the peer-review process, and final publication before drawing any firm conclusions, as others already have concluded (Shankar et al., 2024; Kan et al., 2024).

Those de novo cSDH patients not needing intervention are typically asymptomatic patients with cSDH without mass effect. While observational data tell us that conservative management of these can be successful in the majority of cases – approximately 30% still end up needing surgery (Foppen et al., 2023; Fakhry et al., 2024). Nevertheless, evidence is so far being lacking as to prognostication of the need for intervention in cSDH initially treated conservatively.

Probably the most important aspect of the MMAE technique to consider (if to be used as a stand-alone alternative treatment to surgery) is its temporal efficacy: the duration it takes for the resolution of the mass effect or midline shift after MMAE (Liu et al., 2023). As the MMAE effect is not immediate, MMAE as stand-alone therapy in symptomatic de novo cSDH patients is unlikely to represent optimal care, unless surgery is contraindicated by antithrombotic medication, where the risk of suspending these medications is rarely considered to outweigh the potential benefit of surgery (i.e. those with newly implanted coronary artery stents or metallic heart valves) or those patients with coagulopathy contraindicating surgery. In those rare cases, stand-alone MMAE treatment is most likely the best treatment option. Furthermore, in this context it is important to address the absolute need of surgical intervention in patients with large cSDH at risk of permanent neurological deficits or death if the related mass effect is not urgently addressed. In these cases, surgical decompression (even if associated with significant thromboembolism risk due to the antithrombotics being suspended) is likely to represent the best treatment option. MMAE may however be considered as adjunct to surgery to allow for early resumption of antithrombotics in such situations. These considerations were also reflected upon by the investigators behind the recently finished trials, all of which excluded patients with cSDH who were neurologically unstable and required emergency surgical intervention. Please see Fig. 2.

### 4.2 Use of MMAE in recurrent cSDH

Similarly, to patients with de novo cSDH, recurrent cSDH patients can be roughly subdivided into those needing intervention defined as surgery or MMAE, and those not needing any intervention.

Attempts have been made to predict patients with a higher risk of recurrence. Although characteristics such as large hematoma, low level of consciousness, bilateral hematoma, use of antithrombotics as well as different imaging characteristics on CT and MRI have been suggested as risk factors, the findings reported in the literature are conflicting and based on retrospective datasets (Bartek et al., 2017b). As such, there is currently no reliable way to accurately predict risk of cSDH recurrence. This makes it difficult to robustly determine which patients may benefit from additional treatment options such as MMAE – with the aim of reducing the risk of recurrence at the point of initial diagnosis.

Recurrent surgery in itself carries a substantial clinical and rehabilitative burden for older patients, and patients with recurrence are at risk of long-term functional decline. Consequently, successfully treating the recurrent cSDH patient to avoid further repeated admission, repeated surgery and prolonged hospitalization is of utmost importance. As the recurrence risk after re-do surgery (surgery in recurrent cSDH) is approximately double the recurrence risk following de novo surgery, (approximately 10% risk in de novo surgery and 20% in recurrent cSDH surgery)(Jensen et al., 2022), it seems important to consider minimizing the risk of recurrence, by considering use of MMAE in the context of proven recurrence.

Therefore, in the case of recurrent cSDH, we recommend that MMAE should be considered as adjunctive treatment to surgery, or stand-alone treatment in patients that require intervention but where surgery is prevented due to either coagulopathy, or in those on antithrombotics in whom the risk of suspension is considered unacceptably high. Please see Fig. 2.

### 4.3 Neuroradiological MMAE aspects to consider

Since it's conception, MMAE has (in primarily retrospective studies) shown promising safety and efficacy data (Sattari et al., 2023). Different embolization materials have been used, consisting of microparticles (Polyvinyl-alcohol), liquid embolics, microspheres and coils. In a recent systematic review (Ironside et al., 2021), microparticles were the most frequently used embolization material followed by liquid embolics, coils and microspheres. Currently there is insufficient evidence to support the use of any specific embolic agent.

As to the technical aspects of MMAE, attention must be paid to avoid inadvertent embolization of anastomoses or MMA branches that supply the ophthalmic branch or cranial nerves, as these complications can be devastating (Ironside et al., 2021). When using MMAE in conjunction with surgery, it is currently unclear whether MMAE embolization should be performed before or after surgery (Ironside et al., 2021). It has been suggested that preoperative embolization may be more effective, as middle meningeal artery branches may be injured during surgery, meaning that postoperative embolization could be suboptimal.

### 4.4 Pre-interventional imaging and imaging characteristics

Despite the identification of several imaging characteristics on CT and MRI pointing towards a higher recurrence rate, no clear imaging criteria have been established in the reporting of cSDH.

### 4.5 Post-treatment imaging

One randomized-controlled trial investigated the value of post-treatment CT controls (Schucht et al., 2019). This study found no value of imaging controls after treatment of cSDH. In contrary, CT-controls led to a higher rate of second operations and patients presented a lower 90-day mRS compared to the no-imaging group.

Evidence is currently lacking as to which cSDH hematoma phenotypes should be targeted with MMAE, though hematoma density, membranes character has been considered as prognostic factors for MMAE success (Salem et al., 2023). Similarly, evidence is lacking as to the optimal radiological follow-up routine for cSDH treated with MMAE. In surgically treated cSDH, it has been shown that radiological follow-up is contra-productive and as such has been abandoned by many centres (Schucht et al., 2019), while cSDH cases treated with MMAE typically undergo serial follow-up imaging as routine in most cases.

### 4.6 Perioperative aspects of MMAE care

Aside from strictly surgical or neuroradiological considerations, the decision to proceed to surgery and/or MMAE should take into account anaesthetic concerns, since anaesthesia or sedation is often necessary to allow patients to tolerate the intervention.

The first aspect to consider is the anaesthesiological risk-benefit ratio of surgery as compared to MMAE in the cSDH patients, many of whom are elderly, frail, multimorbid and medicated with antithrombotics (Gaist et al., 2017). Although these patients are vulnerable anesthesiological candidates, MMAE has been shown in observational data to display a better profile in terms of complications and length of hospital stay than surgery (Dowlati et al., 2023). However, an advantage of cSDH surgery is that it can be performed with local anaesthesia and sedation only (Liu et al., 2024), whereas MMAE is frequently performed under general anaesthesia. This is mainly due to decrease the risk of patient movement and to increase patient comfort during longer procedures and during the injection of liquid embolic material (Salem et al., 2024).

An important element of the decision to choose one anaesthetic technique or the other is the effect of unmitigated mass effect of cSDH on the brain, including the presence of elevated intracranial pressure. In such situations, sedation without controlled airway and mechanical ventilation can predispose to airway obstruction, hypoventilation, hypercarbia, and hypoxemia, all of which can be associated with a worsening of intracranial pressure, ischemia, and ultimately additional brain insult (Bonhomme et al., 2009). During general anaesthesia, and particularly in case of elevated intracranial pressure, it is also important to avoid hypotension, which may impede cerebral perfusion pressure and cerebral blood flow (Aguirre et al., 2019). In the absence of general anaesthesia, the risk of movements, seizures, and nausea/vomiting is higher.

Overall, in the context of MMAE, there is currently no evidence to determine superiority of either local or general anaesthesia in regard to complications such as postoperative delirium, mortality, or length of hospital stay (Miller et al., 2018). When deciding on the optimal management, perioperative geriatric assessment can aid in the decision making (Ibitoye et al., 2023). Attention should be paid to provide patients with multimodal analgesia, eventually using locoregional anaesthetic techniques, and to favour opioid-sparing in this older population.

### 4.7 Antithrombotics

In general terms, antithrombotic drugs should be paused prior to surgery, although the thromboembolic risk needs to be balanced with the risk of bleeding (Douketis et al., 2022; Iaccarino et al., 2024). The bleeding risk associated with the surgical removal of cSDH is considered moderate, while it is low for MMAE, or even minimal in the context of radial artery approach (Omura and Ishiguro, 2023). For all patients on antithrombotics, guidelines should be consulted prior to surgery as to decide on the best management strategy (Douketis et al., 2022; Kietaibl et al., 2022).

### 4.8 Post procedure care

Adequate postoperative follow up should ensue, with close attention to analgesia, patient comfort, delirium detection and prevention, and antithrombotic medications management (Samama et al., 2024). The implementation of enhanced recovery programs should also be considered.

### 4.9 Role of geriatric medicine

Although most older patients selected for surgical decompression benefit from decompressive surgery for cSDH, some older people can fail to thrive despite surgery. In such a context, cSDH can represent a sentinel health event, similar to a femoral neck fracture, where co-existent frailty and multimorbidity commonly exist and can drive long term functional decline, especially when complicated by recurrence. Observational data have shown 1-year mortality rate in some patients can be comparable to hip fracture (Miranda et al., 2011). Furthermore, pre-existent frailty has also been demonstrated to be associated with poor functional outcomes following burrhole surgery for cSDH (Kesserwan et al., 2021). Decision making around appropriate management of older people with cSDH can therefore be complex and geriatrician consultation or co-management is advised (Rickard et al., 2023).

Adding to this complexity is the symptomatology of cSDH in older people; they often do not display characteristic focal neurology, but instead present with symptoms of decompensated neurophysiological function, including cognitive impairment, imbalance, impaired ambulation or headache (Blaauw et al., 2022; Bartek et al., 2019). Whilst some healthcare professionals may consider these to represent mild symptoms not warranting intervention, in an older person with physical or cognitive frailty who lacks the physiological reserve to continue independent living, such symptoms may mean the difference between maintaining functional independence and dependence. This not only has significant impact on health economics such as social care costs, but can also greatly affect a person's quality of life. These factors needs to be considered carefully when weighing up the potential management options. In particular it can be important to contemplate what the impact of recurrence would be for an individual already struggling to function without neurophysiological reserve.

Given an acceptable safety profile and apparent efficacy, MMAE has the potential to be a very useful tool in older people. Subgroups in whom MMAE could prove important to consider in future include: a) those whose cSDH (de novo or recurrent) does not meet criteria for burrhole drainage e.g. insufficient clot thickness or midline shift, but whose symptoms are attrributable to cSDH, and which impact, or threaten to impact, on functional independence, b) those who warrant intervention from a hematoma or symptom point of view (de novo or recurrent) but risks of burrhole surgery are considered to be too high, c) those with de novo cSDH in whom a recurrence would be associated with high risk of functional decline e.g. those with mild or moderate frailty where MMAE adjunct might lower the recurrence risk.

Further research is needed to evaluate these hypotheses and therefore these indications remain outside of the scope of advice of this consensus statement. However, in the meantime, neither age nor frailty should be considered barriers to MMAE.

### 4.10 Future directions

It is important to keep in mind, that this consensus statement only reflects the use of MMAE in cSDH, and as such does not reflect upon other management strategies in cSDH.

In the context of MMAE, the panel believes that research efforts should be put into: a) standardized reporting of preoperative symptoms using the Markwalder score and preoperative imaging findings for cSDH, b) stratification and identification of patient subgroups at risk of cSDH recurrence based on clinical and imaging features, and those who might benefit most from MMAE in order to identify which specific treatment options(s) should be used in the different subgroups, c) standardized reporting of treatment- and outcome measures in cSDH in order to facilitate comparison between treatments and to divert attention to alternative endpoints other than recurrence – i.e. functional outcomes such as Modified Rankin Scale or similar (Nasa et al., 2021; Baker et al., 2010), and d) identification of optimal follow-up procedures in patients treated with MMAE. Whilst it has been shown that routine CT controls are not beneficial for surgical candidates (Schucht et al., 2019), evidence for radiological and clinical follow-up routines for MMAE are so far lacking.

Also, we believe that effort should be made to assess the health economic aspect and number needed to treat (NNT) of MMAE in cSDH. Some report on large saving in the form of a -$32,000 lower total 1-year hospital costs if the patient was treated by MMAE + surgery vs. surgery alone (Catapano et al., 2021). However, the healthcare systems differ vastly between Europe and USA and the results have yet to be replicated on other settings. Another way of looking at this is by analysing the NNT. As such, looking at the abstracts from the upcoming 3 RCT trials, one can deduct NNT from the MAGIC-MT and EMBOLISE trial. In the MAGIC-MT trial, the CSDH recurrence rate requiring reoperation after surgery + MMAE was 4.7% vs. 5.2% after surgery only, which adds up to NNT to avoid one reoperation = 200. In the EMBOLISE trial, the CSDH recurrence rate requiring reoperation after surgery + MMAE was 4.1% vs. 11.3% after surgery alone, which adds up to NNT = 14. Nevertheless, we will need to await the publication of these RCTs before drawing any firm conclusions.

Currently, the panel agrees with the recent statement from the ARISE 1 consensus that MMAE does not replace surgical therapy for symptomatic patients (Kan et al., 2024).

Furthermore, perhaps the most important joint agreement of the panel is that the treatment of cSDH needs to be conducted in a patient-centred multidisciplinary approach, with weighing of the opposing risks and benefits of different treatment options in respect to the individual patient. As such, cSDH patients should be managed at institutions where such an approach is achievable.

### 4.11 Limitations

We recognize that this consensus statement has limitations. Firstly, from a methodological point-of-view, although the current level of evidence has been systematically gathered and evaluated by the panel delegates, no Delphi process has been followed when assessing the data and finalising the statement; this was initially deemed unfeasible due to the current heterogeneity in practice between centres (Nasa et al., 2021). Furthermore, at a later stage in the process, it was believed that a Delphi process would add little to the current consensus, as all members of the respective societies could agree a joint statement after the round-table discussion process has been finalised.

Secondly, the GRADE system was also not applied to this process. The GRADE system is for use in guidelines development, which this consensus statement is not, but we also considered that the GRADE system had the potential to add confusion given we were aiming to offer opinion on the integration of more than one intervention: MMAE in the context of stand-alone treatment or as adjunct (Baker et al., 2010). Further, the GRADE system highly relies on randomised data to provide strong recommendations, which further complicates the assessment due to the lack of published randomised data in this context.

Nevertheless, as numerous randomised controlled trials have recently been finalised, with even more on their way, opportunities for guidelines development are expected to present in the future. Moreover, this multidisciplinary panel recognises the need for continued evaluation of the current statement. As such, a new consensus meeting will be required to review the consensus statement in the light of soon to be published trial data.

## 5 Conclusion

The conclusion of this consensus statement is to be considered an expert round table position opinion from representatives of different European and International medical societies involved in the care of cSDH patients. As such, it is a true multi-disciplinary consensus on the role of MMAE in cSDH patients. Interpretation of this statement and its clinical application needs to be conducted by each centre, based on local clinical practice and resources. cSDH remains primarily a surgical disease but should be managed in a multidisciplinary fashion with the involvement of not only the neurosurgeon, but also the neuroradiologist/interventionist, anaesthesiologist and geriatrician. The authors hope that the consensus statement will aid in the development of local treatment protocols for the management of cSDH patients.

Finally, while the role of MMAE in cSDH appears to be promising in the observational data reported, it will be important to review randomised data when it is published. We believe this consensus statement can provide an initial framework to guide clinicians in situations in which it may be considered reasonable to contemplate the use of MMAE in cSDH based on the current available evidence. This requires cautious balancing of early data reporting efficacy against potential complications that are as yet not clearly defined.

## Authors' contributions

Conception and design: J.B., G.C., Z.K., A.R., F.R., T.S., M.M.

Collection and interpretation of data: J.B., A.B., V.B., L.C., F.G., C.I., M.A.A., F.R., C.S.R., T.S., D.S., C.S., M.M.

Manuscript drafting: J.B., V.B., F.G., F.R., T.S., D.S., M.M.

Manuscript editing: J.B., A.B., V.B., L.C., G.C., M.C., G.D.N., E.D.R., F.G., C.I., Z.K., M.A.M., A.R., F.R., C.S.R., T.S., D.S., C.S., M.M.

Manuscript approval for submission: J.B., A.B., V.B., L.C., G.C., M.C., G.D.N., E.D.R., F.G., C.I., Z.K., M.A.M., A.R., F.R., C.S.R., T.S., D.S., C.S., M.M.

## Funding

The Consensus Meeting was arranged and supported with the non-conditioning assistance of BALT. The EANS delegates were not financially supported by BALT, the expenses of these were reimbursed by the EANS.

## Declaration of competing interest

The authors declare the following financial interests/personal relationships which may be considered as potential competing interests: Jiri Bartek and all authors received editorial assistance and project facilitation support from Alison Crowe as declared in the manuscript reports administrative support was provided by Alison Crowe at corvus communications limited. Alessandra Biondi reports a relationship with Balt, Medtronic, Microvention, Penumbra and Stryker Neurovascular that includes: consulting or advisory and funding grants. Vincent Bonhomme reports a relationship with Medtronic, Edwards Medical, Orion Pharma, Grünenthal, and Elsevier as Deputy Editor-in-Chief of the Acta Anaesthesiologica Belgica that includes: consulting or advisory and funding grants. Lucio Castellan reports a relationship with Roche, Biogen, Stryker, VMLY Seagen, Seagen, Balt and H2O that includes: consulting or advisory, funding grants, and speaking and lecture fees. Corrado Iaccarino reports a relationship with Integra Life Science and Finceramica S.p.A. that includes: funding grants. Zsolt Kulcsar reports a relationship with Microvention, Stryker, Medtronic and Cerenovus that includes: consulting or advisory and funding grants. Markus Moelenbruch reports a relationship with Balt, Medtronic, Microvention, Stryker that includes: funding grants. Andreas Raabe reports a relationship with Zeiss that includes: consulting or advisory and funding grants. Carolina S Romero reports a relationship with Octapharma that includes: funding grants. David Shipway reports a relationship with Balt that includes: travel reimbursement. Carmine Sicignano reports a relationship with Balt that includes: speaking and lecture fees. Mario Muto reports a relationship with Stryker that includes: consulting or advisory. The Consensus Meeting was arranged and supported with the non-conditioning assistance of BALT. The EANS delegates (Jiri Bartek, Marco Cenzato and Andreas Rabbe) were not financially supported by BALT, the expenses of these were reimbursed by the EANS. If there are other authors, they declare that they have no known competing financial interests or personal relationships that could have appeared to influence the work reported in this paper.
